# Comparing the Effect of Group and Individual Mindfulness‐Based Cognitive Behavior Therapy on Happiness of Postmenopausal Women: A Randomized Controlled Trial in Iran

**DOI:** 10.1002/hsr2.70649

**Published:** 2025-04-16

**Authors:** Basad Terikani, Parvin Abedi, Maryam Gholamzadeh Jofreh, Maryam Dastoorpour, Poorandokht Afshari

**Affiliations:** ^1^ Midwifery Department, Menopause Andropause Research Center Ahvaz Jundishapur University of Medical Sciences Ahvaz Iran; ^2^ Department of Counseling, Ahv.C. Islamic Azad University Ahvaz Iran; ^3^ Department of Biostatistics and Epidemiology, School of Public Health Ahvaz Jundishapur University of Medical Sciences Ahvaz Iran

**Keywords:** happiness, mindfulness cognitive behavioral therapy, post menopause

## Abstract

**Background and Aims:**

Happiness is an important factor for decreased quality of life in some postmenopausal women. This study aimed to compare the effects of group and individual mindfulness‐based cognitive behavioral therapy (MBCT) on the happiness of postmenopausal women.

**Methods:**

This was a randomized controlled trial of 48 postmenopausal women who received group or individual MBCT counseling. Literate postmenopausal women aged 50–64 years, 1–10 years after menopause, and with Happiness Scale scores less than 40–42 were recruited for this study. A demographic questionnaire and the Oxford Happiness Questionnaire were used to collect the data. Women in the group counseling group received 8 sessions of counseling according to MBCT, while women in the individual counseling group received counseling individually. Independent *t*‐tests, Chi‐square tests and two‐way Repeated Measures ANOVA were used to analyze the data.

**Results:**

The scores of all components of happiness including self‐concept, life satisfaction, mental preparation, active well‐being, aesthetic sense, self‐efficacy, and hope, increased in both groups immediately after and 1 month after the completion of the intervention. The total happiness score in the group counseling group improved from 33.39 ± 5.57 before the intervention to 48.91 ± 6.77 and 48 ± 6.38 after the intervention and during the follow‐up period, respectively (*p* value < 0.001). These scores in the individual counseling group improved from 32.13 ± 5.40 to 46.04 ± 7.80 and 45.13 ± 7.26 immediately after the intervention during the follow‐up period, respectively (*p* value < 0.001). There was no significant difference between the two groups.

**Conclusion:**

Our results showed that although the scores of all components of happiness were slightly greater in the counseling group than the individual group, there was no significant difference between the two groups. Using either method is recommended for increasing the happiness of postmenopausal women.

**Abbreviations**
ANOVA: Analysis of variance
BDI‐II: Beck Depression Inventory
CBT: Cognitive behavioral therapy
MBCT: Mindfulness‐based cognitive behavioral therapy
OHI: Oxford Happiness Index

## 1 Introduction

Menopause defined as the permanent cessation of menstruation due to a lack of estrogen that occurs around the age of 50 years in most women [1]. This natural biological process is often accompanied by adverse physiological and psychological effects. Vasomotor symptoms, vaginal atrophy, osteoporosis, and cardiovascular disease are examples of adverse physical effects, while sleep disturbances, anxiety, and low mood are examples of psychological consequences of menopause [2]. These multifaceted challenges can contribute to reduced self‐care behaviors among postmenopausal women [3].

Happiness, an important factor in quality of life, also decrease in some postmenopausal women due to the low level of estrogen and consequences of menopause. Life satisfaction is one of the components of happiness that may deteriorate during the post‐menopausal period. In their study, Patucka et al. reported that postmenopausal women have moderate life satisfaction [4]. Additionally, in a systematic review including 14 articles and 6293 postmenopausal women, Sharifi et al. reported that the quality‐of‐life scores were moderate and needed to be improved [5].

Psychological interventions have shown promise in addressing these challenges. For instance, Sadeghi et al. demonstrated that group‐based happiness training significantly improved psychological capital encompassing hopefulness, resilience, optimism, and self‐efficacy among postmenopausal women [6].

One of the methods that may improve the psychological symptoms of menopause is mindfulness‐based cognitive behavioral therapy (MBCT). MBCT, introduced by Segal et al. is a method that combines cognitive behavioral therapy (CBT) with meditation and mindfulness (focusing on present without judgment) [7]. Studies have shown that MBCT can significantly enhance psychological well‐being and emotional regulation in postmenopausal women [8, 9]. Additionally, physical activity and group discussions have been found to improve happiness and quality of life in this population [10].

Despite the growing evidence supporting the benefits of MBCT, the optimal delivery format group versus individual remains underexplored. Group‐based interventions offer unique advantages, such as social support, shared experiences, and cost‐effectiveness, which may enhance therapeutic outcomes [11]. On the other hand, individual counseling allows for personalized attention and tailored interventions, which may be particularly beneficial for addressing specific psychological needs [12]. However, limited research has directly compared the efficacy of group and individual MBCT in improving happiness among postmenopausal women.

Given the importance of happiness and its impact on quality of life, this study aims to compare the effects of group and individual MBCT on the happiness of postmenopausal women. By examining these two delivery formats, we seek to identify the most effective approach for enhancing psychological well‐being in this population. We hypothesize that both group and individual MBCT will significantly improve happiness scores, with potential differences in the magnitude or nature of their effects.

## 2 Materials and Methods

### 2.1 Design

This was a parallel randomized controlled trial of 48 postmenopausal women who received group or individual mindfulness cognitive behavior therapy counseling.

### 2.2 Inclusion/Exclusion Criteria

Literate postmenopausal women aged 50–64 years, 1–10 years after menopause, and with Happiness Scale scores less than 40–42 were recruited for this study.

Women with known physical or psychological disorders, who were taking psychological medications, who were abused of drugs, who had experienced major stressor events in the past 3 months, and who had participated in counseling classes during the past year were excluded from the study. We ruled‐out depression using the Beck Depression Inventory II (BDI), and according to the BDI‐II, only women with no depression or mild depression were recruited. The criteria for drop‐out were if women not participated in two sessions of counseling.

### 2.3 Sample Size

Considering a previous study [13] and using the following formula the sample size was calculated to be 19 in each group. Considering a 20% probability of withdrawal, the final sample size was calculated to be 24 in each group. The variable used to calculate the sample size was happiness.

$$n=\frac{(s_{1}^{2}+s_{2}^{2}){\left(z_{1-\frac{\alpha }{2}}+z_{1-\beta }\right)}^{2}}{{\left({\bar{x}}_{1}-{\bar{x}}_{2}\right)}^{2}}$$

Z_1 − α/2_ = 1.96
Z_1 − β_ = 1.64
Mean_1_ = X_1_ = 62.81
Mean_2_ = X_2_ = 53.79
Standard deviation1 = S1 = 8.78
Standard deviation1 = S2 = 5.81
*n* = (8.78^2^ + 5.81^2^) (1.96 + 1.64)^2^/(62.81 + 53.79)^2^ = 111(13)/81 = 19

### 2.4 Randomization and Allocation Concealment

Eligible women were randomized into two groups using the block randomization with a block size of four and an allocation ratio of 1:1. The code dedicated to each group was written in a piece of paper and was kept with the Secretory of a Public Health Center. Therefore, neither the researcher nor the participants were aware of the grouping until the commencement of the intervention. Due to the nature of this study, blinding was not possible. The data collector (BT) was not blinded, but the statistician was not aware of grouping.

### 2.5 Instruments

A demographic questionnaire and the Oxford Happiness inventory (OHI) were used to collect the data. Additionally, the Beck's Depression Inventory (BDI‐II) was used to assess depression among participants.

The demographic questionnaire was consisted of questions about age, duration of menopause, marital status, educational attainment, number of children, occupation, economic status, age and educational status of the husband. This questionnaire was approved by seven faculty members in the Department of Midwifery.

The OHI was developed by Hills and Argyle at Oxford University in 2002 [14]. This questionnaire has 29 questions, and each question is scored from zero (strongly disagree) to 3 (strongly agree).

This questionnaire has seven subscales including self‐concept (8 questions), life satisfaction (4 questions), mental preparation (4 questions), active well‐being (2 questions), aesthetic sense (5 questions), self‐efficacy (4 questions), and hope (2 questions). The minimum and maximum scores of this questionnaire vary from zero to 87.

The psychometric evaluation of the Persian version of this questionnaire was approved by Alipour et al [15]. Their results showed that the internal consistency of OHI was appropriate and all 29 items were highly correlated with the total score. The Cronbach's alpha for the questionnaire was 0.91. The Pearson's correlation coefficient between the OHI and the BDI and Extraversion and Neuroticism of EPQ subscales were respectively −0.48, 0.45, and −0.39, confirming their convergent and divergent validity.

The BDI‐II has 21 multiple‐choice questions that measure the feelings of participants during the past 2 weeks. Each question was scored from zero (stands for better feeling) to three (stands for unbearable sadness or unhappiness). The minimum and maximum scores of this questionnaire ranged from 0 to 63, with higher scores indicating worse depressive feelings. Scores of 0–21 indicate mild depression, 21–42 indicate moderate depression, and scores > 42 indicate severe depression [16]. The Persian version of this questionnaire was evaluated and approved by Tanjani et al. in Iran [17]. In the present study only women with no depression or mild depression were recruited.

### 2.6 Procedure

The women in the group counseling were classified into two groups of 12 and received eight sessions of counseling using mindfulness‐based cognitive behavioral therapy on a weekly basis. Women receiving face‐to‐face counseling received eight sessions of the same counseling individually. Each session lasted 90 min. The content of the sessions is illustrated in Table 1 [18]. All counseling sessions were conducted by the lead author (Basad Terikani) under the supervision of Dr. Maryam Gholamzadeh Jofreh. This author has studied counseling in midwifery and has attended relevant workshops to learn MBCT.

**Table 1:** Content of eight sessions of counseling.

Sessions	Content	Homework
First	In this session, the researcher firstly introduced herself. Then she stated the rules and goals of the sessions and established a therapeutic relationship. She explained menopause and its effect on life, and taught practicing body inspection, starting the exercise focusing on breathing, and giving feedback. The researcher explained homework, and provided audio files containing the texts of soothing and happy sounds and pamphlets of the first session for the participants.	Body inspection and focus on breathing
Second	In this session, the participants practiced body inspection exercise and reviewed their exercise and homework. Then, they were taught how to manage thoughts and feelings (walking on the street), recording pleasant events, and sitting meditation for 10–15 min. Then, the researcher distributed handouts of the second session and asked the participants to present their homework.	Listening to the file of “body check” every day per week, mindful breathing for presence of mind 10–15 min every day, recording pleasant or enjoyable events (every day) mindfulness in normal activities.
Third	In this session, the participants were requested to practice the following: seeing or hearing, sitting meditation, practicing 3‐min breathing space, practicing mindful walking and practicing mindful lying down and reviewing these exercises. The participants were instructed to review the homework of the second session.	Breathing and stretching exercises in the first, 3rd, and 5th day of the week, practicing movement with a conscious state of mind on the second, fourth, and sixth days of the week, recording a report of experiencing an unpleasant event, analyzing this unpleasant event and making it pleasant, practicing 3 min of breathing space three times a day.
Fourth	The participants were requested to have 5 min of visual or auditory mindfulness, meditation in a sitting position (awareness of breathing, body, sounds, thoughts, and awareness without specific direction). The researcher then introduced this method as a coping strategy to be used at times of difficult emotional conditions. The participants learned to have a 3‐min breathing space. Then, they reviewed the homework of the third session.	Meditation in a sitting position, a 3‐min breathing space according to the usual routine (three times a day), a 3‐min breathing space as a coping strategy (during times of experience unpleasant feelings) and continue to repeat the technique until a pleasant feeling is created
Fifth	In this session participants were taught the following topics: awareness of breathing and body, emphasis how to react to thoughts, feelings and bodily sensations. Then they performed session exercises as follows: meditation in the created sitting posture, introducing a difficult position in the exercise and exploring its effects on the body and mind, and 3 min of breathing space. The participants then reviewed the assignments of the previous session, and the researcher distributed the pamphlets of the current session and set the assignments of the session.	Meditation in a sitting position, 3 min of breathing space (three times every day), 3 min of breathing space as a coping strategy (when experiencing unpleasant feelings) and repeating the technique until they felt good.
Sixth	In this session, the participants were requested to do the following: Meditation in the sitting position, awareness of breathing and the body, talking about happiness, as well as joyful and happy events, repetition of happy sentences and getting familiar with the exercise and realizing its effects on the body and mind, 3 min of breathing space, and reviewing the assignments of the fifth session.	40 min of daily practice, working with a different combination of the three main exercises, gaining awareness using a range of shorter exercises, gaining awareness through doing exercises with and without using 3 min of breathing space, 3 min of breathing space.
Seventh	In this session, the participants were requested to do the following: Meditation in a sitting position, awareness of breathing, body, listening to soothing and happy sounds, 3 min of breathing space problem solving or correcting problems, correcting techniques to find out its effects on the body and mind, and reviewing the assignments of the last session.	Choosing a model from all different types of exercises that can be pursued after the end of the program. Breathing space a routine and as a coping strategy, the practice of discovering a way to open the door to perform skillful action, developing an early warning system to detect a relapse, developing an action plan that can be used in the face of low mood.
Eight	The participants were requested to do the following: Thinking about physical examination and finishing it, reviewing the homework of the previous session and choosing a program for homework that can be continued until the next month.	

Women requested to complete demographic and BDI‐II questionnaires. Then, women with moderate or severe depression were excluded and referred to a psychologist. Women with no depression or mild depression were requested to complete the OHI before the intervention, and those with scores less than 40–42 were recruited for the intervention. Participants in both groups requested to complete the OHI before the intervention, immediately after the intervention and 1 month after the completion of the intervention.

### 2.7 Ethical Considerations

The Ethics Committee of Ahvaz Jundishapur University of Medical Sciences approved the design of the study (Ref No: IR. AJUMS. REC.1402.255). The study protocol was registered in the Iranian Registry for Randomized Controlled Trials (Ref No: IRCT20230724058911N1, registration date: 21/8/2023). All participants provided written informed consent before data collection. The anonymity of information gathered from participants was observed.

### 2.8 Statistical Analyses

Descriptive statistics, including frequency distribution, mean, and standard deviation, were employed in this study. The Shapiro‐Wilk test was utilized to assess the normality of the data. To evaluate the homogeneity of variables such as age, duration of menstruation, spouse's age, number of children, education level, spouse's education, marital status, employment status, and economic status between the two groups at baseline, the independent two‐sample *t* test and Chi‐square test were utilized.

To examine the temporal changes in response variables and analyze both within‐group and between‐group effects, the two‐way repeated measures ANOVA was utilized. The study specifically investigated the effect of the group variable (group intervention, individual intervention), the time variable (pre‐intervention, post‐intervention, follow‐up period), and the interaction effect between group and time. Post‐hoc tests using Tukey's method in the repeated measures ANOVA model were conducted to examine the specific differences between groups and time points following a significant result from the two‐way Repeated Measures ANOVA.

Two‐sided tests were used, and a significance level of less than 0.05 was considered. Data analysis was performed using SPSS software version 16.

## 3 Results

**Figure 1:**
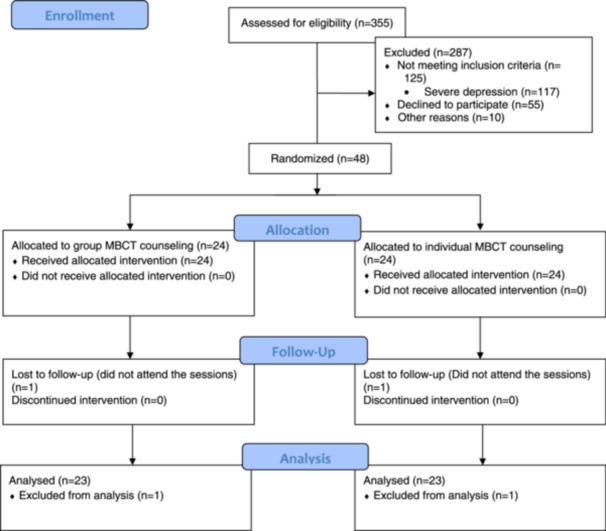
CONSORT flow diagram of recruitment and retention of participants in the study.

We recruited 24 women in each group. One woman in each group dropped out. The reason for drop‐out are given in Figure 1. The demographic characteristics of the participants in the two groups receiving group counseling and individual counseling are presented in Table 2. As evident from this table, the mean ages of the participants were (mean ± standard deviation) 57.26 ± 3.61 and 57 ± 4.36 years in the group and individual counseling, respectively. Approximately 6 years passed from menopause in the two groups. Most participants in group counseling had a diploma (9(52.94%)) while those in the individual group had a university education or high school (7(38.89%). Most women in the two groups were married and housewives (22(95.65%), and had moderate economic status [19 (82.61%) and 16 (69.57%) in the group and individual counseling respectively]. There were no significant differences in demographic characteristics between the two groups.

**Table 2:** Demographic characteristics of participants in the two groups receiving group and face‐to‐face counseling.

Variables	Group counseling *n* = 23	Individual counseling *n* = 23	*p* value
	Mean ± SD		
Age (y)	57.26 ± 3.61	57 ± 4.36	0.826
Age of husband (y)	63.94 ± 4.57	60.55 ± 7.41	0.203
Years passed from menopause (y)	6.74 ± 3.40	6.26 ± 3.52	0.642
Number of children	3.13 ± 1.74	3.22 ± 1.08	0.840
	*N* (%)		
**Education**			
High school	9 (39.13)	10 (43.48)	0.615
Diploma	12 (52.17)	9 (39.13)	
University education	2 (8.69)	4 (17.39)	
**Marital status**			
Married	17 (73.91)	18 (78.26)	> 0.999
Single	2 (8.69)	1 (4.35)	
Widow	4 (17.39)	4 (17.39)	
**Occupation**			
Employed	1 (4.35)	1 (4.35)	0.756
Housemaker	22 (95.65)	22 (95.65)	
**Economic status**			
Weak	1 (4.35)	5 (21.74)	0.285
Moderate	19 (82.61)	16 (69.57)	
Good	3 (13.04)	2 (8.69)	

Table 3 was designed to compare the scores of the components and total score of happiness before and after the intervention and during the follow‐up period. As this table shows, the two groups did not show any significant differences in self‐concept before the intervention, while this score increased immediately after and during the follow‐up period in both groups. Although this increase was slightly greater in the group counseling group than in the individual counseling group, the difference was not significant. Life satisfaction was increased significantly in both groups after the intervention and during the follow‐up period. Although this increase was slightly greater in the individual group than in the group counseling group, the differences between the two groups were not significant.

**Table 3:** Comparison of components of happiness among the two groups of group and face‐to‐face counseling before and after the intervention.

Variables	Group counseling Mean ± SD	Within group		Individual counseling Mean ± SD	Within group		Between groups		*p* value‐Effect of group	*p* value‐Effect of time	*p* value‐The group‐time interaction effect
**Self‐concept**		MD (95% CI for MD)	*p* value		MD (95% CI for MD)	*p*‐value	MD (95% CI for MD)	*p* value			
Before	8.70 ± 2.40	—	—	8.91 ± 2.13	—	—	0.22 (−1.08–1.52)	0.741	0.577	< 0.001	0.059
After	12.91 ± 2.31	4.22 (3.67–4.76)	< 0.001	12.17 ± 2.21	3.26 (2.72–3.81)	< 0.001	−0.74 (−2.04–0.56)	0.263			
Follow‐up	12.35 ± 2.15	3.65 (3.11–4.20)	< 0.001	11.83 ± 2.17	2.91 (2.37–3.46)	< 0.001	−0.52 (−1.82–0.78)	0.429			
**Life satisfaction**											
Before	4.78 ± 1.20	—	—	4.61 ± 1.59	—	—	−0.17 (−0.90–0.55)	0.638	0.927	< 0.001	0.689
After	7.17 ± 1.03	2.39 (1.93–2.85)	< 0.001	7.22 ± 1.45	2.61 (2.15–3.07)	< 0.001	0.04 (−0.68–0.77)	0.906			
Follow‐up	6.78 ± 0.95	2.00 (1.54–2.46)	< 0.001	6.83 ± 1.15	2.22 (1.76–2.68)	< 0.001	0.04 (−0.68–0.77)	0.906			
**Mental preparation**											
Before	5.78 ± 1.54	—	—	5.26 ± 1.86	—	—	−0.52 (−1.46–0.43)	0.281	0.051	< 0.001	0.315
After	7.70 ± 1.64	1.91 (1.33–2.49)	< 0.001	5.57 ± 1.65	1.30 (0.73–1.88)	< 0.001	−1.13 (−2.08–0.17)	0.021			
Follow‐up	7.26 ± 1.60	1.48 (0.90–2.06)	< 0.001	6.39 ± 1.50	1.13 (0.55–1.71)	< 0.001	−0.87 (−1.82–0.08)	0.074			
**Active well‐being**											
Before	2.17 ± 1.15	—	—	1.74 ± 1.01	—	—	−0.44 (−1.13–0.26)	0.220	0.564	< 0.001	0.270
After	3.09 ± 1.28	0.91 (0.46–1.36)	< 0.001	3.17 ± 1.37	1.43 (0.98–1.89)	< 0.001	0.09 (−0.61–0.78)	0.806			
Follow‐up	3.44 ± 1.12	1.26 (0.81–1.72)	< 0.001	3.26 ± 1.21	1.52 (1.07–1.98)	< 0.001	−0.17 (−0.87–0.52)	0.623			
**Aesthetic sense**											
Before	5.70 ± 1.66	—	—	5.57 ± 1.80	—	—	−0.13 (‐1.01–0.75)	0.770	0.392	< 0.001	0.551
After	8.13 ± 1.58	2.43 (1.89–2.98)	< 0.001	7.78 ± 1.20	2.22 (1.67–2.76)	< 0.001	−0.35 (‐1.23–0.53)	0.437			
Follow‐up	8.17 ± 1.59	2.48 (1.93–3.02)	< 0.001	7.65 ± 1.15	2.09 (1.54–2.63)	< 0.001	−0.52 (‐1.40–0.36)	0.244			
**Self‐efficacy**											
Before	3.96 ± 1.26	—	—	3.74 ± 1.01	—	—	−0.22 (‐1.1–0.67)	0.627	0.191	< 0.001	0.242
After	6.39 ± 1.59	2.44 (1.97–2.90)	< 0.001	5.74 ± 1.71	2.00 (1.54–2.46)	< 0.001	−0.65 (‐1.53–0.23)	0.146			
Follow‐up	6.39 ± 1.50	2.44 (1.97–2.90)	< 0.001	5.65 ± 1.85	1.91 (1.45–2.38)	< 0.001	−0.73 (‐1.62–0.14)	> 0.999			
**Hope**											
Before	2.30 ± 0.77	—	—	2.30 ± 0.70	—	—	0.00 (0.00–0.00)	> 0.999	0.732	< 0.001	0.772
After	3.52 ± 0.85	1.21 (0.89–1.54)	< 0.001	3.39 ± 0.99	1.09 (0.76–1.41)	< 0.001	−0.13 (‐0.62–0.36)	0.601			
Follow‐up	3.61 ± 0.78	1.30 (0.98–1.63)	< 0.001	3.52 ± 0.95	1.22 (0.89–1.54)	< 0.001	−0.09 (‐0.58–0.41)	0.728			
**Total score of happiness**											
Before	33.39 ± 5.57	—	—	32.13 ± 5.40	—	—	−1.26 (‐5.10–2.58)	0.517	0.208	< 0.001	0.266
After	48.91 ± 6.77	15.52 (13.91–17.14)	< 0.001	46.04 ± 7.80	13.91 (12.30–15.53)	< 0.001	−2.86 (‐6.71–0.97)	0.142			
Follow‐up	48 ± 6.38	14.61 (12.99–16.22)	< 0.001	45.13 ± 7.26	13.00 (11.39–14.62)	< 0.001	−2.86 (‐6.71–0.97)	0.142			

*Note:* The scores of self‐concept, mental preparation, aesthetic sense, self‐efficacy, and hope.

Abbreviation: MD, mean difference.

After the intervention, mental preparation increased during group counseling and during the follow‐up period. Although this difference was significant at each of the two time points, when we evaluated the effect of time and group together, it was not significant.

The score of “active well‐being” increased slightly in both groups, but the two groups did not show any significant difference regarding this issue. The scores for “aesthetic senses” improved more in the group counseling group than in the individual group, but the differences between the two groups were not significant. Self‐efficacy and hope improved more in the group counseling group than in the face‐to‐face group immediately after the intervention and during the follow‐up period, but after considering the effect of time, these differences did not remain significant. The total happiness score in the group counseling improved from 33.39 ± 5.57 before the intervention to 48.91 ± 6.77 and 48 ± 6.38 after the intervention and during the follow‐up period, respectively (*p* value < 0.001). These scores improved in the individual group counseling from 32.13 ± 5.40 to 46.04 ± 7.80 and 45.13 ± 7.26 immediately after the intervention and during the follow‐up period, respectively (*p* value < 0.001). Although the differences between the two groups were significant at the two time points, they did not remain significant after adjusting for the effect of time (*p* value = 0.2655).

## 4 Discussion

This study aimed to compare the effect of group MBCT with individual counseling on the happiness of postmenopausal women. Our results showed that the scores of all components of happiness including self‐concept, life satisfaction, mental preparation, active well‐being, aesthetic sense, self‐efficacy, and hope, increased in the two groups of face‐to‐face and group MBCT counseling. Although the scores of all components of happiness including mental preparation, self‐concept, aesthetic sense, self‐efficacy, hope, and total score of happiness were slightly greater in the group counseling than in the individual group, there was no significant difference between the two groups after considering the effect of time and group.

The mechanism by which MBCT can improve happiness may be related to the following factors: 1. Cognitive reprogramming and emotional regulation: MBCT helps individuals examine their thoughts, identify unhelpful patterns, and regulate emotions. This process involves mindfulness practices that encourage present‐moment awareness and non‐judgmental acceptance of thoughts and feelings [18]. 2. Neurobiological changes: MBCT may promote changes in brain regions associated with emotional regulation, such as the hypothalamus. These changes can enhance reward‐seeking behaviors, reduce avoidance behaviors, and improve emotional well‐being [19].

3. Social and psychological benefits: Happiness is closely linked to social bonding, goal attainment, and psychological well‐being. MBCT can facilitate these outcomes by improving self‐awareness, self‐efficacy, and hope, which are critical components of happiness [20, 21].

Although both group and individual MBCT have been studied in various studies, these two methods have differences, as follows: 1. Social support and shared experiences: The group setting provides a unique opportunity for social interaction and shared experiences. Participants can benefit from peer support, normalization of their experiences, and collective learning. This social dynamic can enhance mental preparation and emotional resilience, as evidenced by the significant improvement in mental preparation in the group setting compared to individual counseling [22]. While individual counseling offers personalized attention and tailored interventions, it lacks the social reinforcement and shared learning that group settings provide. This may explain why all components of group MBCT in our study showed greater improvement after intervention.

Another difference of group and individual MBCT is normalization and validation. Being part of a group can help participants feel less isolated in their struggles. Hearing others' experiences and realizing that they are not alone can validate their feelings and reduce stigma, which can indirectly boost happiness. Furthermore, in individual settings, the therapist's validation is crucial, but it may not carry the same weight as peer validation in a group setting [23].

Finally, another difference between group and individual MBCT is dynamics of learning and practice. Group settings often involve interactive exercises, group discussions, and collective mindfulness practices. These activities can enhance engagement and motivation, making it easier for participants to integrate mindfulness into their daily lives, but individual sessions may focus more on personal challenges and specific mindfulness techniques tailored to the individual's needs. While this can be highly effective, it may lack the communal energy and accountability that group settings provide [24].

We could not find any study that compare the group and individual MBCT on happiness of postmenopausal women. However, Khoshbooi et al. in their study on 64 depressed perimenopausal Iranian women found that 15 sessions of culturally adapted Cognitive Behavioral Therapy in group or individual could significantly improve depression and sexual satisfaction of perimenopausal women [25]. Also, Rezaei et al. in their study on 96 postmenopausal women who assigned into two groups of in person and virtual counseling found that both methods could improve quality of life of postmenopausal women. It is worth mentioning that women in both groups classified into groups with 10–12 participants [26]. Our results in terms of effect of group or individual counseling are similar to above‐mentioned studies.

Some studies have assessed the effect of counseling programs using CBT or MBCT on happiness in different groups. For example, a study by Bakalim showed that 8 weeks of counseling could significantly improve positive emotions, happiness and life satisfaction among university students [27]. Another study by Dhami et al. showed that 16 weeks of CBT in patients with depression could significantly replace event‐related potentials for angry stimuli with happiness stimuli, and this effect was proven by electroencephalography [28]. Our results are in line with those of the above‐mentioned studies.

In line with our study, Talebi et al., in a study to evaluating the effect of 8 weeks of MBCT on anxiety, happiness, and mindfulness in nurses, found that although this treatment could increase happiness and mindfulness, it failed to decrease anxiety [29]. Our results are in line with those of Talebi et al., except for that in the current study, we did not measure anxiety, but we recruited women who did not have depression or who had mild depression.

Additionally, Skolzkov et al., in their study of 83 psychology students, found that MBCT could decrease depression and increase subjective happiness. Although they found positive results in their study, they concluded that to obtain better results, the meditation should be continued [30].

A study in Iran among university students at Kerman University of Medical Sciences showed that 8 sessions of MBCT increased the scores of emotional, psychological, and social well‐being among students and decreased the scores of depression, anxiety, and stress [31]. Our results are in line with those of the above‐mentioned studies.

## 5 Limitations of the Study

This is the first study in which we compared the effect of two counseling methods, group and individual MBCT, on the happiness of Iranian postmenopausal women. Despite its merits, this study has several limitations. First, the reports of participants about their home works and their responses to the questionnaires may have been affected by recall bias. Second, although not having depression or mild depression was our inclusion criterion, we did not measure anxiety or stress, which may have affected the results of the study. Third, blinding was not possible in this study, and this may affect the results of the study. Finally, the psychological preparedness of women in the group counseling was certainly different, and this may affect the results of the study. However, we tried to present the counseling in simple and understandable language.

## 6 Conclusion

The results of this study showed that although scores of all components of happiness were slightly greater in the counseling group than in the individual group, there was no significant difference between the two groups. Both methods significantly improved all components of happiness as well as the total happiness score. Using either method is recommended for increasing the happiness of postmenopausal women.

## Author Contributions

**Basad Terikani:** conceptualization, investigation, methodology, writing – review and editing, data curation, project administration. **Parvin Abedi:** conceptualization, investigation, funding acquisition, methodology, supervision, resources, writing – review and editing, writing – original draft. **Maryam Gholamzadeh Jofreh:** conceptualization, investigation, methodology, supervision, writing – review and editing. **Maryam Dastoorpour:** conceptualization, investigation, methodology, writing – review and editing, software, formal analysis, supervision. **Poorandokht Afshari:** conceptualization, investigation, methodology, writing – review and editing, supervision, validation.

## Ethics Statement

The Ethics committee of Ahvaz Jundishapur University of Medical Sciences approved the design of the study (Ref No: IR. AJUMS. REC.1402.255). The protocol of the study was registered in the Iranian Registry for Randomized Controlled trial (Ref No: IRCT20230724058911N1).

## Consent

All participants provided written informed consent before data collection. The anonymity of information gathered from participants were observed.

## Conflicts of Interest

The authors declare no conflicts of interest.

### 1 Transparency Statement

The corresponding author (PA) affirms that this manuscript is an honest, accurate, and transparent account of the study being reported; that no important aspects of the study have been omitted; and that any discrepancies from the study have been explained.

## Data Availability

The data that support the findings of this study are available from the corresponding author upon reasonable request. The data of this study will be available upon reasonable request from the corresponding author.
